# Synergy of Art, Science, and Technology: A Case Study of Augmented Reality and Artificial Intelligence in Enhancing Cultural Heritage Engagement

**DOI:** 10.3390/jimaging11030089

**Published:** 2025-03-19

**Authors:** Ailin Chen, Rui Jesus, Márcia Vilarigues

**Affiliations:** 1 Department of Conservation and Restoration, NOVA LINCS, NOVA School of Science and Technology, 2829-516 Caparica, Portugal; ailin.chen@outlook.com; 2 Lisbon School of Engineering (ISEL), Polytechnic University of Lisbon, NOVA LINCS, 1959-007 Lisbon, Portugal; 3 Department of Conservation and Restoration, VICARTE, NOVA School of Science and Technology, 2829-516 Caparica, Portugal; mgv@fct.unl.pt

**Keywords:** cultural heritage, artificial intelligence, augmented reality, hyperspectral imaging

## Abstract

In recent years, there has been growing interest in taking advantage of the technological progress in information technology and computer science to enhance the synergy between multidisciplinary organisations with a mutual objective of improving scientific knowledge and engaging society in cultural activities. Such an example of collaboration networks includes those where governmental, scientific and cultural institutions work in unison to provide services that support research through the use of technology while disseminating information and promoting cultural heritage. Here, we present a case study implementing the results of the work between multidisciplinary departments of the NOVA University Lisbon and third-party cultural heritage organisations. In particular, a mobile and desktop PC application uses augmented reality to showcase results obtained from analysis of artwork by Amadeo de Souza-Cardoso using artificial intelligence. The mobile application is intended to be used to enhance museum visitors’ experience and strengthen the link between scientific, governmental, and heritage organisations.

## 1. Introduction

As we continue striding in the 21st century with rapid advancement and innovation in multitudinous aspects, digitalisation is no longer a new concept referring to technological development but has become more of a lifestyle. Society 5.0 is the very example of such a revolution in terms of digitalisation, where humans are deemed the centre of our society. This idea was first introduced in Japan, following a chronological definition of Hunting and Gathering Society (Society 1.0), Farming Society or Agricultural Society (Society 2.0), Industrial Society (Society 3.0), Information Society (Society 4.0), and Human-Centred Society (Society 5.0) [1,2,3].

Society 5.0 implements state-of-art technologies such as Artificial Intelligence (AI) and the Internet of Things (IoT) to facilitate day-to-day human life. The majority of these applications in Society 5.0 focus on public services such as healthcare and transportation along with daily activities such as eating, shopping, and learning. Sometimes, the focus is extended to broader services such as disaster prediction and prevention, agricultural optimisation, and logistics automation, among others [1,2,3]. However, relatively less attention has been paid to the cultural heritage sector in Society 5.0, specifically the implementation of high technology in artistic settings such as galleries and museums.

Applications of Augmented Reality (AR) and Virtual Reality (VR) that can provide an interactive and immersive experience for users have been gradually introduced to visitors in so-called cultural heritage tourism over the last few years [4]. Studies concerning AR and VR have been slowly established to evaluate the impact on a user-centred experience. For instance, the work in [5] investigated the use of AR at a Jewish museum targeting mainly school children, and reported a more engaging and enhanced learning experience in an educational and cultural context as well as more sustainable museum infrastructure. The ArkaeVision project [4] introduced a game-like virtual environment to further motivate users towards “culturally-qualified” immersion in a storytelling context to explore historical architectures. This approach was deemed to be very promising and to provide a communicative and explorative approach to an educational and cultural heritage learning experience. The work in [6] created a large-scale VR experience juxtaposing the virtual and physical spaces, making full use of human physical, tactile, and vestibular sensations via stimulation from visual and audio information provided by VR. Such installations have proven to be very convenient; they are scalable, tourable, can be easily moved around without conventional fixated installations, and are reapplicable in different museums. In the work of [7], the author intended to conduct a direct comparison of user experiences between these two types of technologies. The acquired results showed positive feedback in both areas around enjoyment, presence, cognitive, and emotional and behavioural engagement, with slightly higher scores obtained for VR technology than AR technology in only a few aspects. The work in [8] presented a case study that confirmed the value of AR in economic, social, cultural, and historical aspects from stakeholders’ perspectives. The study published in [9] also reviewed different systems, including AR, VR, and mixed-reality systems, in order to identify appropriate installation for specific cases in cultural heritage settings. Other examples include the work in [10], where two AR applications were tested and the study determined that active interactive participation improved participants’ understanding of historical events, and the work in [11], where experiments were performed on school children by integrating educational learning with AR games with the goal of making understanding and learning more entertaining.

Despite the continuous advancement and emergence of AR, VR, and mixed-media systems, their use in cultural and historical environments is still sparse [12]. In Portugal, the use of these technologies in the areas of authentication, conservation, and restoration remains limited. In the pursuit of bridging the gap between cultural heritage and technology, an AR application and desktop PC application are proposed based on research artworks by the late Portuguese artist Amadeo de Souza-Cardoso. Their implementation and use is expected to help with the dissemination of information via more engaging and captivating methods for museum and gallery participants. Similarly, their use is intended to help connect the collaborative networks around governmental entities, the scientific community, and cultural institutions. This paper elaborates on the details concerning the application’s development by a successfully established collaborative network and its benefit in the pursuit of a Society 5.0 environment.

## 2. Background

We present a case study that showcases some of the results collated from a long-term project established in the field of cultural heritage conservation and restoration. In particular, this work is focused on artwork by the late Portuguese artist Amadeo de Souza-Cardoso. The developed application is intended to analyse his artwork while creating tools that facilitate the scientific and forensic analysis of his creations. Through this project, it is expected that the results will lead to a more thorough understanding of this artist’s style and techniques as well as to the implementation of processes for art authentication and prevention of forgeries. The acquired knowledge and information will also contribute to the production of techniques and material for the dissemination of information and the inclusion of society in the preservation of cultural heritage.

**Figure 1:**
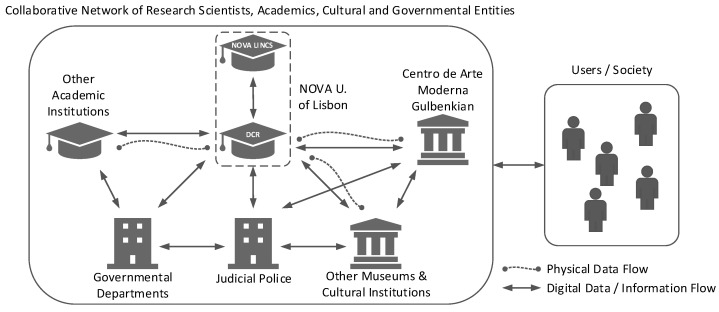
Block diagram of the collaborative network involved in the case study.

The successful progress of the project to date has been achieved by the establishment of a network of organisations sharing a common objective, namely, the study and dissemination of information related to cultural heritage. as shown in Figure 1, the structure of this collaborative network contains a variety of entities, including scientific researchers from multiple academic institutions, government departments, historians, curators, and museums.

During the earlier stages of the project [13,14], a dynamic team effort between entities was required in order to complete the data acquisition process. This included invaluable assistance from the museums for direct access to the artwork along with the support of internal and external academic laboratories for access to specialised analytical and hyperspectral imaging equipment. In addition to the use of temporary virtual laboratories for physical data processing, the majority of the team’s interactions were conducted through the use of digital channels for sharing digital information, methodologies, and results along with the organisation of other activities.

In subsequent stages of the project, researchers from different areas of specialisation turned their attention to the analysis of the data by employing analytical and digital processing techniques. This interdisciplinary work resulted in the generation of algorithms to determine features of interest in the artwork, e.g., chemical composition, colour identification, and degree of authenticity. These algorithms were implemented in a desktop PC application to facilitate analysis of data, in particular by government institutions seeking to verify the authenticity of works of art. The results and information collated by these scientific, cultural, and government organisations can then be used by the judicial authorities to police and enforce the protection of national cultural heritage.

With the information and progress attained in this manner, the focus of the research team turned to the next step, where the following questions were considered. First, how can the knowledge and techniques developed in this case study be used for the benefit of society? Second, is it possible to increase social awareness of the need to preserve cultural heritage while engaging society through the use of modern technology?

## 3. Methodology

Addressing the proposed research questions requires a comprehensive, multidisciplinary, and collaborative approach primarily led by cultural organisations. To begin exploring these questions, the research team proposed the development of a desktop and mobile application designed for the analysis and visualisation of artwork. The application would be available to both skilled professionals and the general public. The particular use case scenario targeted society at large, where it was envisaged that museum visitors could use an AR-enabled mobile application to enhance their visiting experience. The expected outcome of this solution was twofold: first, that the mobile application would be used to disseminate results obtained by the research group; and second, that it would engage users with art through technology, thereby fulfilling the objective of benefiting society with a step forward towards Society 5.0.

### 3.1. Amadeo AR: Overview

**Figure 2:**
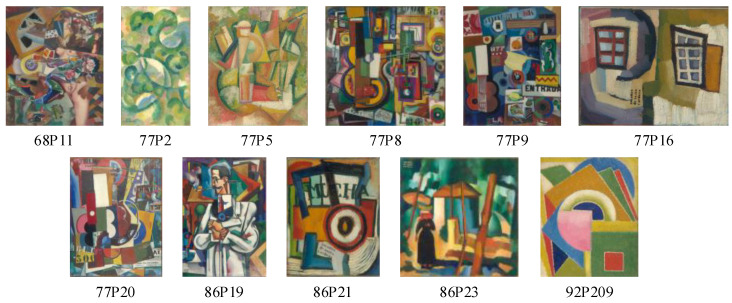
Overview of the 11 evaluated paintings by Amadeo de Souza-Cardoso. Painting images © Center of Modern Art of Calouste Gulbenkian Foundation, Lisbon, Portugal.

The applications examined in this study focus on the artwork of Amadeo de Souza-Cardoso, primarily displayed at the Calouste Gulbenkian Museum in Lisbon, Portugal. The artwork was limited initially to a group of 11 paintings of Souza-Cardoso (Figure 2) along with a selection of 16 pigments known to have been used by the artist. A group of individuals from different organisations were involved in the temporary acquisition of the original artwork from the Calouste Gulbenkian Museum, Lisbon, Portugal, for photography and digitisation. The resulting hyperspectral images were subsequently made available to the research team for further analysis.

The artworks were analysed using hyperspectral and analytical data obtained via the implementation of deep learning algorithms developed by the research team. These algorithms were deployed in a desktop PC application called the Amadeo Image Analysis Tool, and incorporated the following two primary methods:

- A combination of numerical metric functions and three-dimensional convolutional neural networks for image segmentation and classification of pure pigments [15].
- Estimating the probability of authorship of the artwork based on brushstroke analysis using convolutional neural networks [16].

After postprocessing, The first method generated results that produced images highlighting individual pigments within the artworks. These images were subsequently compiled into a database destined for visualisation, ideally within an AR environment. The proof of concept for Amadeo AR was developed at the NOVA University of Lisbon using imagery obtained through painting analysis. To maximise accessibility, the mobile application was developed using the Android operating system, given its significant global market share of 71% compared to the 28% of its main competitor, iOS [17].

**Figure 3:**
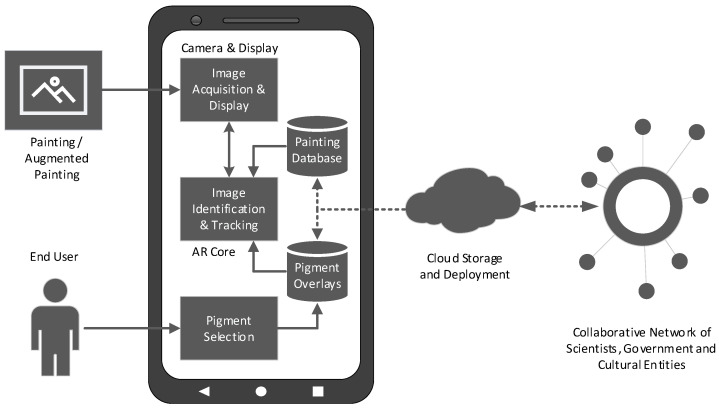
Block diagram of Amadeo AR. Dotted lines indicate proposed future development features.

The primary functionality of the mobile application is currently limited to the visualisation of the previously identified features through the use of AR technology. Figure 3 presents the block diagram outlining the data structure and functionality of Amadeo AR.

The Amadeo AR visualisation process involves capturing and displaying frames in real-time using the device’s integrated camera. These frames are analysed using Google’s ARCore technology to identify paintings from a predefined reference database. The identified images are then augmented by overlaying visual data that highlight key features, namely, the pure pigment distribution. These overlay images are generated through machine learning algorithms and hyperspectral image analysis conducted by the research team, and are stored locally on the device.

**Figure 4:**
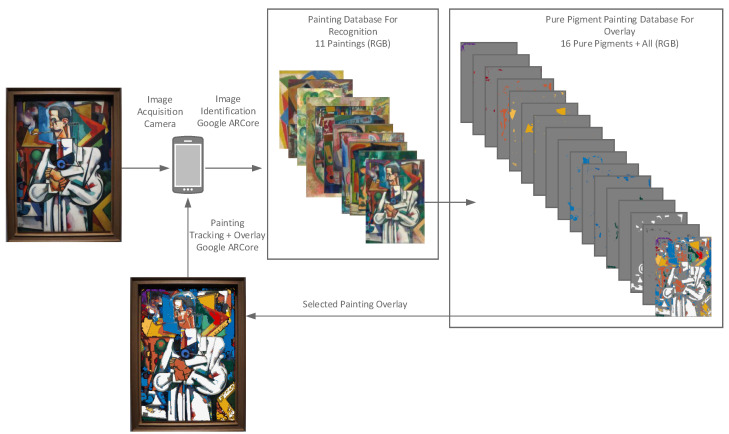
Detailed diagram of image data in Amadeo AR.

At present, the mobile application is limited to the use of reference and overlay images derived from the 11 paintings analysed from the Souza-Cardoso collection (Figure 4). However, future integration of cloud services is expected to enable real-time access to newly analysed images, which will expand the application’s dataset. This advancement will allow the research team to continuously enhance the repository of analysed artworks, potentially facilitating the inclusion of works from other artists and genres.

### 3.2. AmadeoAR: Usage

This section details the functionality of the Amadeo AR application, demonstrating its use with images of paintings by Amadeo de Souza-Cardoso. It provides examples and practical instructions for operation on a mobile device.

**Figure 5:**
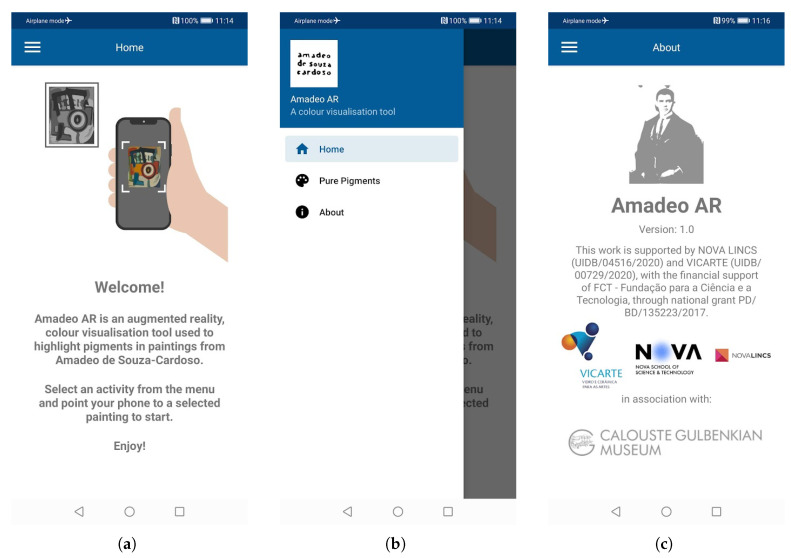
Amadeo AR main screens: (**a**) Welcome, (**b**) Menu, and (**c**) About.

Upon launching the application, the user is greeted with a welcome screen (Figure 5a). A menu button is available to show the currently available activities: “Home”, “Pure Pigments”, and “About” (Figure 5b). Selecting “Home” returns the user to the “Welcome” screen. Selecting “About” directs them to a page detailing basic information about the project and application, including software version, sponsors, and the associated institution, in this case the Calouste Gulbenkian Museum, Lisbon, Portugal (Figure 5c).

**Figure 6:**
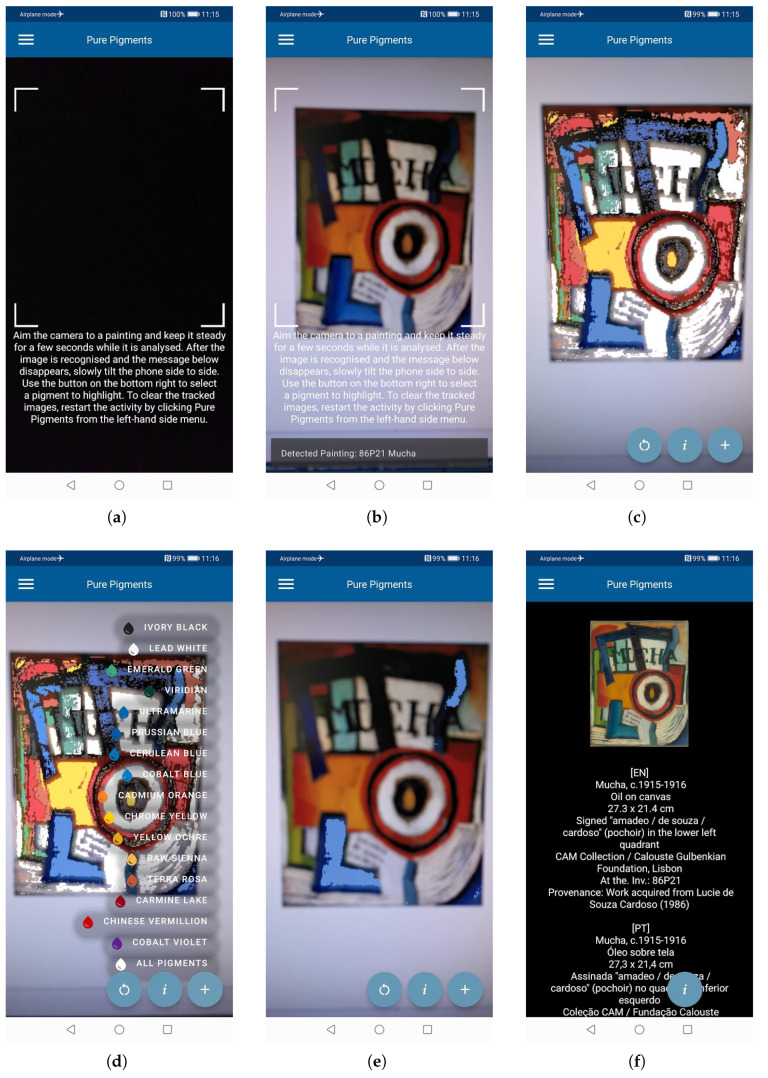
Amadeo AR “Pure Pigments” example screens, showing the process of tracking an image and overlaying pure pigments: (**a**) instructions, (**b**) image aiming, (**c**) image overlay, (**d**) list of available overlays, (**e**) single overlay, and (**f**) painting information.

By clicking “Pure Pigments”, the user is directed to the primary activity containing a live-camera screen where images of Souza-Cardoso’s paintings can be scanned (Figure 6a). The user is required to point the camera towards a painting and keep the mobile device steady in order to allow the software to scan and recognise the image successfully (Figure 6b). If the image of the painting is recognised, the predicted pure pigment overlay image is displayed floating on top of the original image (Figure 6c). The user is then presented with three buttons: a “reload” symbol, “i”, and “+”. The “+” button shows the list of all available pure pigments, allowing the user to select a single (Figure 6e) or all pigments (Figure 6d) to be highlighted. The “i” button brings up a screen with information related to the currently tracked painting (Figure 6f). Finally, the “reload” button refreshes the screen and clears the AR image so that the user can start afresh to scan a new painting image.

### 3.3. Amadeo Image Analysis Tool: Overview

The Amadeo Image Analysis Tool is a desktop application designed to implement the algorithms developed in this research. Thus, the deep learning models and processing are tailored to suit the artwork of Amadeo de Souza-Cardoso. However, its modular architecture allows for easy adaptation and expansion to include works by other artists.

**Figure 7:**
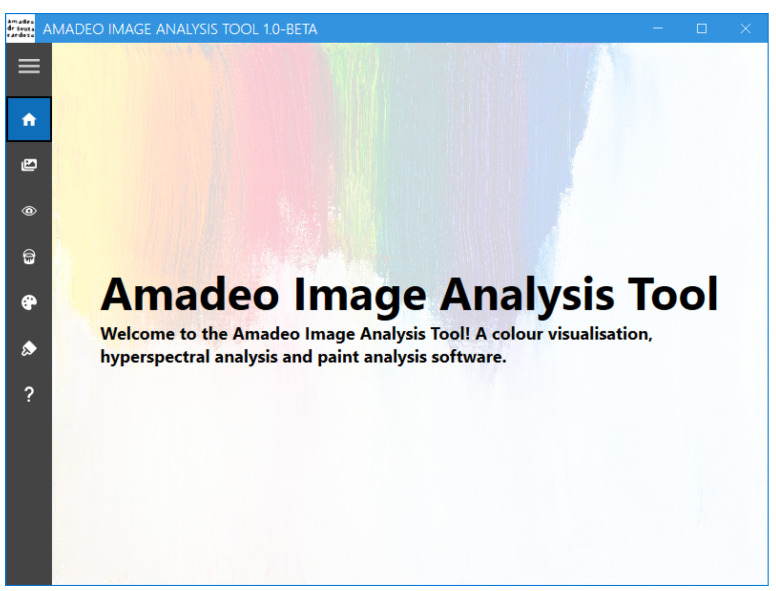
Amadeo Image Analysis Tool: *Home* screen.

Currently, the Amadeo Image Analysis Tool (Figure 7) offers the following functionalities:

- Conversion of sRGB images to hyperspectral data [18]
- Hyperspectral image visualisation
- Pure pigment prediction and visualisation [15]
- Reference pigment palette visualisation
- Brushstroke-based artwork authentication [16]

The conversion of sRGB images to hyperspectral images uses artificial neural networks to estimate the reflectance of an image on a pixel-by-pixel basis. The pure pigment prediction module applies deep learning to identify regions in an artwork that closely match pigments from a reference palette. Additional comparison methods included to evaluate spectral similitude are RMSE, SAM, and SCM. Brushstroke analysis employs a deep learning model to assess the likelihood of a given painting being an authentic work of Souza-Cardoso based on its stylistic characteristics.

### 3.4. Amadeo Image Analysis Tool: Usage

This section provides an overview of the Amadeo Image Analysis Tool interface and instructions for its use, illustrated with examples from Souza-Cardoso’s paintings.

**Figure 8:**
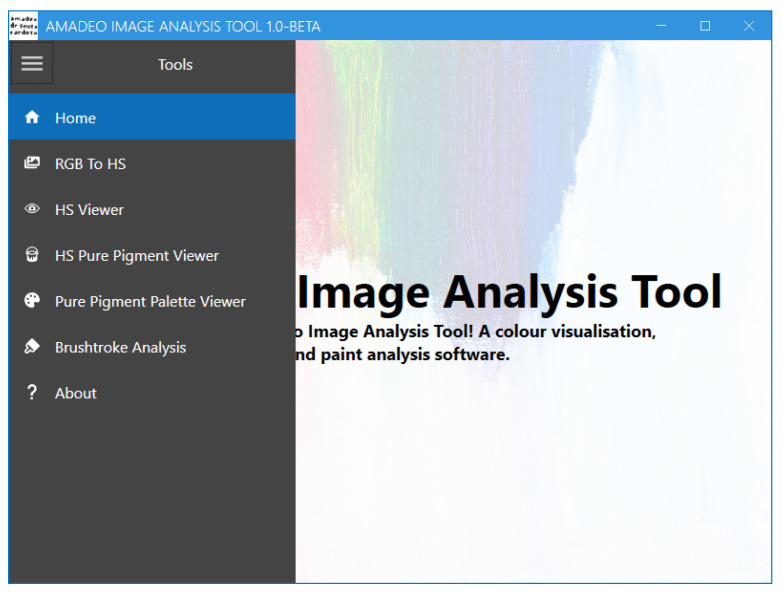
Amadeo Image Analysis Tool: Menu screen.

The tool features a navigation menu that provides the user access to the multiple screens that are available, as shown in Figure 8. These include “Home”, “RGB To HS”, “HS Viewer”, “HS Pure Pigment Viewer”, “Pure Pigment Palette Viewer”, “Brushstroke Analysis”, and “About”.

**Figure 9:**
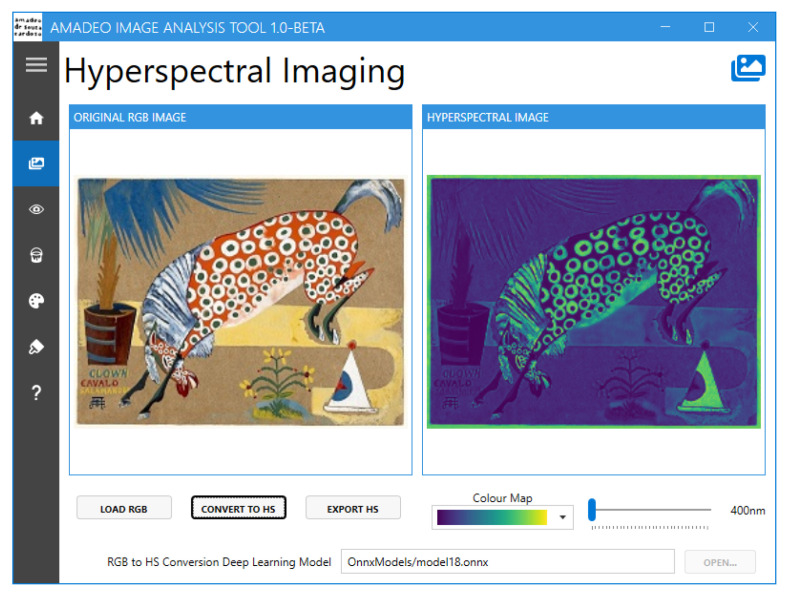
Amadeo Image Analysis Tool: “RGB to HS” screen.

The “RGB To HS” screen (Figure 9) allows the user to load an sRGB image in BMP, PNG, or JPG format and convert it into hyperspectral data using a deep learning model. The user can visualise the estimated hyperspectral image at multiple wavelengths with various colour maps and export the data as an image, animated GIF, or Matlab file (version 9.13).

**Figure 10:**
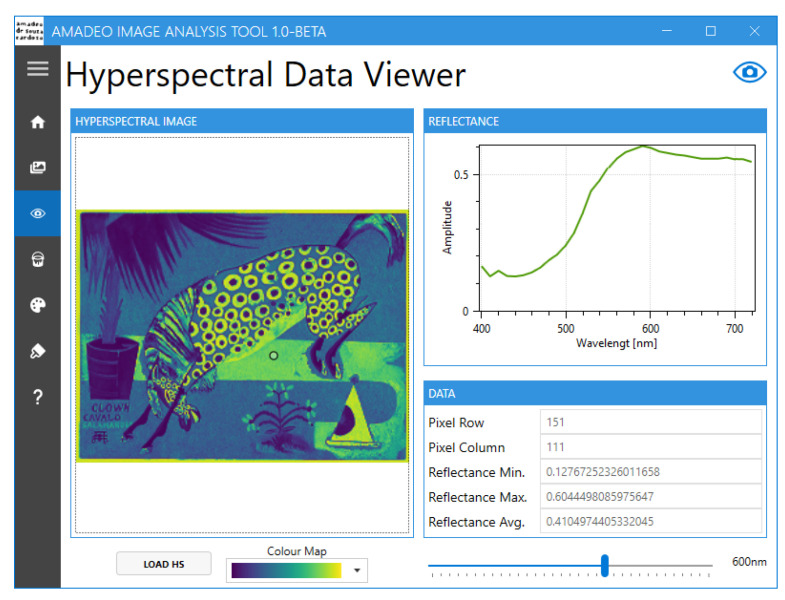
Amadeo Image Analysis Tool: Hyperspectral Data Viewer screen.

In the “HS Viewer” screen (Figure 10), the user can visualise the reflectance of any point in a hyperspectral image by clicking on the image. This hyperspectral image can be the image estimated in the previous screen. Alternatively, the user can load a Matlab file containing the hyperspectral image. Zoom functionality is provided for ease of use.

**Figure 11:**
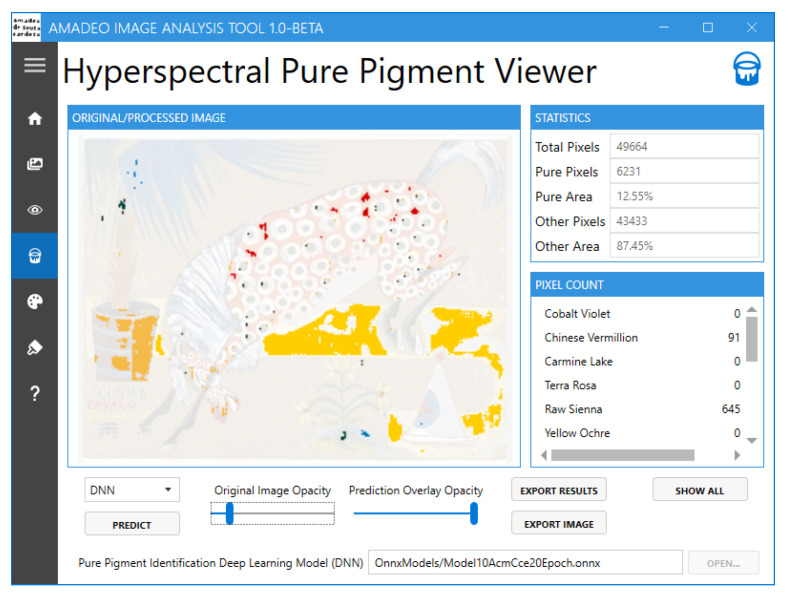
Amadeo Image Analysis Tool: “HS Pure Pigment Viewer” screen.

The “HS Pure Pigment Viewer” screen (Figure 11) allows the user to predict the pure pigments in the currently loaded hyperspectral image using a deep learning model (DNN) or one of the available metric functions SCM, SAM, or RMSE. On the right-hand side, information concerning the detected pure pigments is shown. At the bottom of the screen, the user can choose either the opacity of the original image or the opacity of the predicted overlay, allowing them to analyse the exact location of the detected pure pigments in more detail. When the user is satisfied with the comparison between the original sRGB image and the image with the predicted pure pigments and has tweaked the opacity of each image as desired, they can use the “EXPORT RESULTS” button to export the results in a CSV file or the “EXPORT IMAGE” button to export the results to an image file.

**Figure 12:**
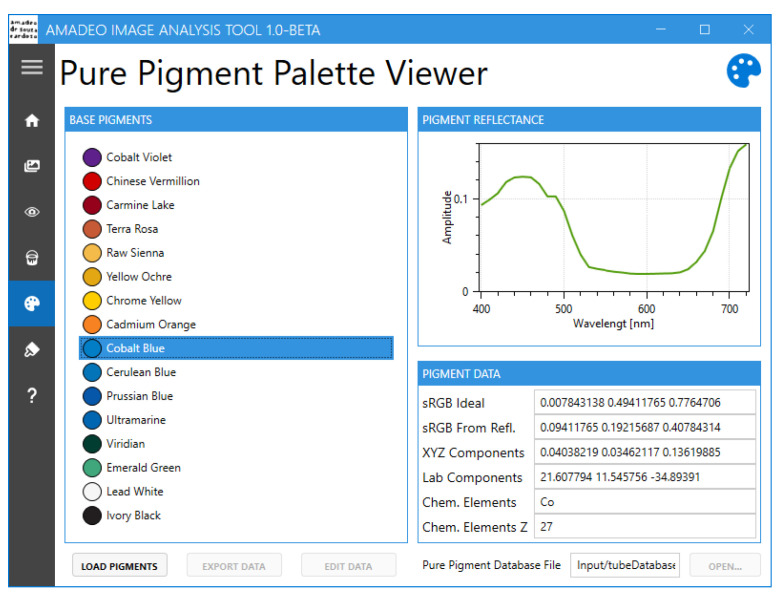
Amadeo Image Analysis Tool: “Pure Pigment Palette Viewer” screen.

Choosing “Pure Pigment Palette Viewer” (Figure 12) allows the user to view information about the pure pigments used in this project. The available information includes the spectral curve of each pigment, the colour coordinates in the sRGB, XYZ, and CIELab colour components, and the relevant chemical components associated with each pure pigment. The 16 default pure pigments are the pigments obtained directly from the paint tubes that Souza-Cardoso used during his lifetime. Theoretically, the user can also load other pure pigment database files if available.

**Figure 13:**
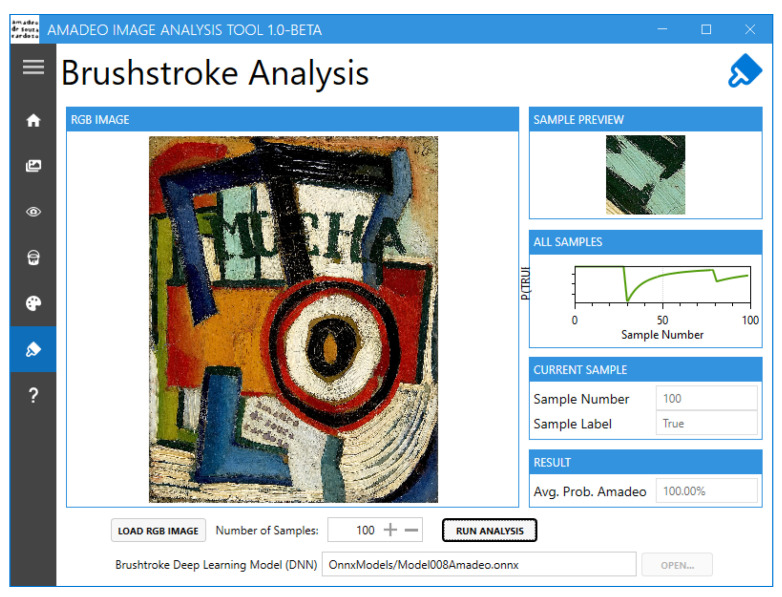
Amadeo Image Analysis Tool: “Brushstroke Analysis” screen.

In the “Brushstroke Analysis” screen (Figure 13), the user can analyse an sRGB image of a painting using a deep learning model to predict whether or not the painting is an authentic artwork by Souza-Cardoso. First, the user must load the sRGB image of the painting to be analysed in BMP, PNG, or JPG format, then select the number of samples of the image to be used for analysis. A higher number of samples will lead to the result being more accurate. The size of the samples used in the algorithm is 227×227 pixels, corresponding to randomly cropped images from the painting image; these samples are augmented as described in [16]. Upon clicking “RUN ANALYSIS”, the progress and results will be displayed on the right-hand side of the screen, where individual samples can be previewed. When the processing is finished, the final probability in terms of the authenticity of the processed painting belonging to Souza-Cardoso is displayed at the bottom.

**Figure 14:**
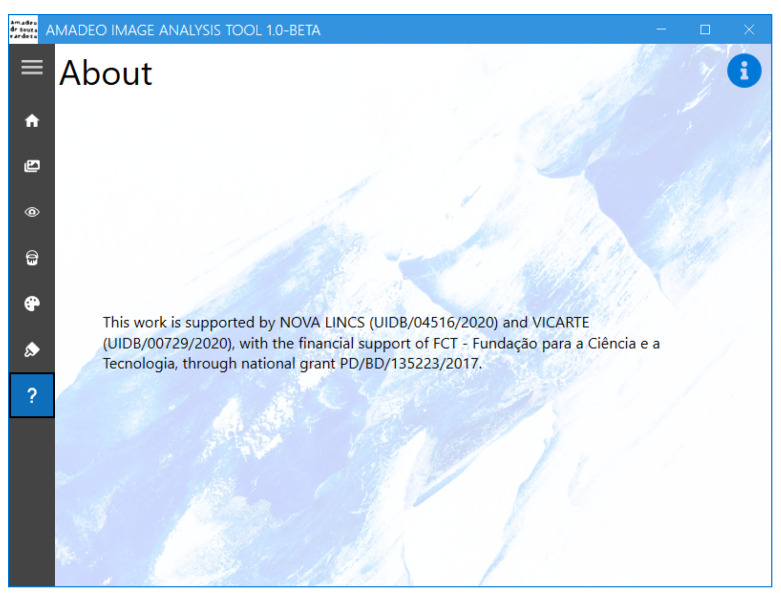
Amadeo Image Analysis Tool: “About” screen.

Finally, the “About” screen (Figure 14) contains information regarding the sponsor of the work and the developed application.

## 4. Results: Usability Trial

**Figure 15:**
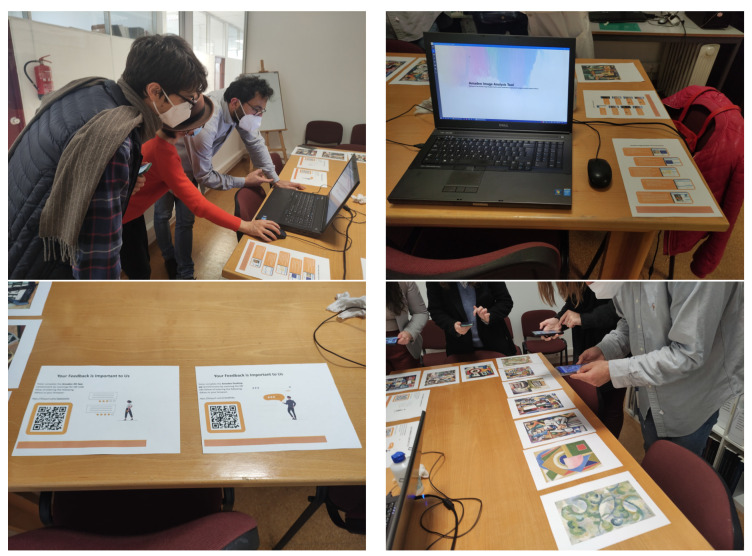
Usability trials of Amadeo AR and Amadeo Image Analysis Tool at DCR, NOVA University Lisbon.

Usability trials were conducted to evaluate both the Amadeo Image Analysis Tool (desktop application) and the Amadeo AR mobile application. The desktop application was assessed as a tool for scientific researchers and governmental organisations, while the mobile application was designed to address the research questions posed in this study. However, due to the unforeseen circumstances of the COVID-19 pandemic, user trials and experience-based questionnaires could only be conducted within the Department of Conservation and Restoration at NOVA University Lisbon (Figure 15).

To ensure participant engagement and minimise response fatigue, the questionnaires were designed to be concise, simple, and easy to complete using Google Forms. The key findings from these trials are summarised below.

**Figure 16:**
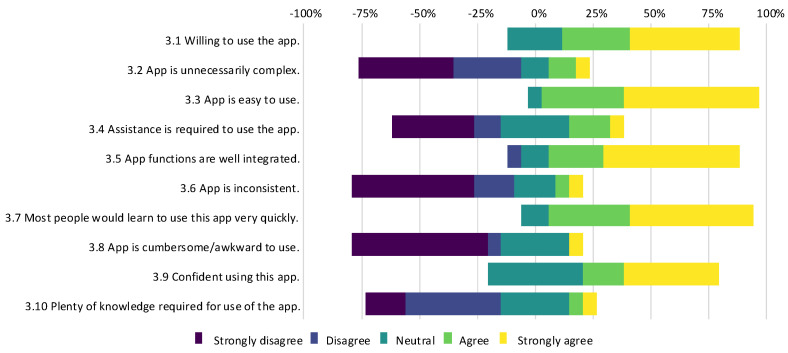
Survey results for questions 3.1 to 3.10 from the desktop PC application trial.

A group of 17 participants from the scientific community were provided access to the desktop application along with sample digital images of artworks for evaluation. They subsequently submitted feedback via a structured digital questionnaire. The results shown in Figure 16 suggest that although the majority of the participants found the desktop PC application easy to use, some improvements to make the application more intuitive would be beneficial. For example, these could include adding help buttons, descriptive text, and tutorials. Overall, the application was well received by the academic community, as indicated by one participant’s comment: “*Add another analysis like Raman, but it was perfect until now and I’m surprised*.”

**Figure 17:**
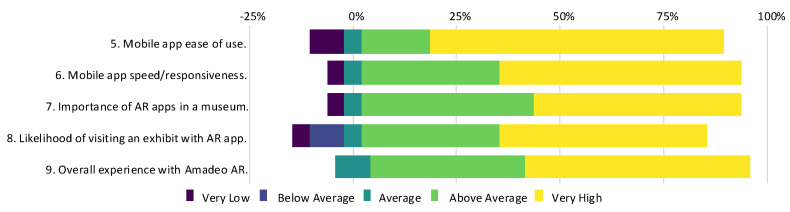
Survey results for questions 5 to 9 from the mobile application trial.

The Amadeo AR application was evaluated by 24 volunteers from diverse backgrounds. Participants tested the functionality of Amadeo AR using printed reproductions of the artwork and provided feedback through an online survey. The findings of the survey revealed that 79.2% of the participants were unfamiliar with Amadeo de Souza-Cardoso or his artwork, despite his significance as a modernist artist in Portugal. This finding lends strength to the need for initiatives that engage society with local heritage and promote cultural activities. Additionally, 91.7% of participants reported no prior experience with AR applications, meaning that their feedback can provide an unbiased assessment of user satisfaction. The majority of participants reported a positive experience, as shown by the results in Figure 17. Furthermore, when asked about the importance of AR applications in cultural settings such as museums and whether AR technology would increase their likelihood of visiting exhibitions, the vast majority responded affirmatively. Less than 5% of respondents indicated low importance, reinforcing the potential of AR tools in enhancing museum visitor experience. The findings of the trial in the case study support the proposed solution as a needed tool for bridging the gap between technology and cultural heritage. The mobile Amadeo AR application was particularly well received, highlighting its potential as a tool for fostering engagement with art and heritage.

## 5. Conclusions and Further Work

The presented case study demonstrates the successful implementation of a solution for information dissemination of cultural heritage and engagement of society in cultural activities. The success of the project was driven by a multidisciplinary collaborative network of research scientists, museums, and governmental institutions. The collective expertise, knowledge, and resources shared by this team allowed the project to overcome challenges related to the collection and digitisation of data, sharing of digital information, development of algorithms, and implementation of the Amadeo AR mobile application.

The results of the usability trials confirmed the suitability of AR applications as a tool for distributing information and engaging society in activities involving cultural heritage. Further work is planned to include other artworks by Souza-Cardoso. In addition, it is envisaged that the developed methodology will be used to analyse additional artists and genres. To this end, further collaboration with other museums and research institutions will be established. Finally, future application development will include the use of cloud services to store and deploy up-to-date information on the available works of art. This will transform the Amadeo AR application into a comprehensive and publicly accessible digital resource for cultural heritage.

## Data Availability

The data presented in this study are available upon request from the corresponding author, subject to copyright restrictions and permission from the relevant sources.

## Abbreviations

The following abbreviations are used in this manuscript:

IOT	Internet of Things
AR	Augmented Reality
VR	Virtual Reality
sRGB	Standard Red, Green, Blue
RMSE	Root Mean Square Error
SAM	Spectral Angle Mapper
SCM	Spectral Correlation Mapper
HS	Hyperspectral
BMP	Bitmap format
JPG	Joint Photographic Experts Group format
PNG	Portable Network Graphics format
CSV	Comma-Separated Values
DNN	Deep Neural Network learning model
